# Supplementary material for: Real-time simultaneous refractive index and thickness mapping of sub-cellular biology at the diffraction limit
Source: Commun Biol. 2024 Feb 6;7:154. doi: 10.1038/s42003-024-05839-w (PMC10847501; doi:10.1038/s42003-024-05839-w)
Supplement: Supplementary file 2 — Supplementary information [file 42003_2024_5839_MOESM2_ESM.pdf]

Real-time simultaneous refractive index and thickness mapping of sub-cellular biology at the diffraction limit – Supplementary material

Arturo Burguete-Lopez[1], Maksim Makarenko[1], Marcella Bonifazi[1,2], Barbara Nicoly Menezes de Oliveira[1], Fedor Getman[1], Yi Tian[1], Valerio Mazzone[1,2], Ning Li[1], Alessandro Giammona[3,4], Carlo Liberale[3], Andrea Fratalocchi[1*]

[1]*PRIMALIGHT, Computer, Electrical and Mathematical Sciences and Engineering (CEMSE), King Abdullah University of Science and Technology (KAUST), Thuwal 23955-6900, Saudi Arabia*

[2]*Physik-Institut, University of Zurich, Winterthurerstrasse 190, Zurich 8057, Switzerland*

[3]*Biological and Environmental Science and Engineering Division (BESE), King Abdullah University of Science and Technology (KAUST), Thuwal, 23955-6900, Saudi Arabia.*

[4]*Institute of Molecular Bioimaging and Physiology (IBFM), National Research Council (CNR), Segrate, Italy*

[*]*andrea.fratalocchi@kaust.edu.sa*

Supplementary Note 1

Analogously to how the cones in the human visual system translate a spectra into three different stimulus values, the camera translates the reflection spectra received by each sensor pixel into three color signals (RGB). This mapping takes place for each channel independently through the operation $X = \int_{\lambda_0}^{\lambda_f} P(\lambda)x(\lambda)\,\mathrm{d}\lambda$, where $P(\lambda)$ is the observed SPD, $X = \{R, G, B\}$ is one of the color channels, and $x(\lambda) = \{r(\lambda), g(\lambda), b(\lambda)\}$ is one of the color matching functions (CMF's) of the camera.

The CMFs are related to the sensitivity of the sensor pixels to light and the nature of the color filters placed before them. While for the human visual system the CMF's are well known, due to manufacturing differences each camera has its own set of CMF's. We may approximate each CMF for a given camera as a linear combination of basis functions so that $x(\lambda) = \sum_{n=0}^{\infty} k_n b_n(\lambda)$ with $b_n(\lambda)$ being the basis and k_n being a set of coefficients.

In this work we carry out the expansion using Gaussian functions as the basis. The choice follows from the assumption that the camera CMFs should look similar to the human visual system CMFs, but other functions such as Lorentzian curves or sinusoids can be employed for the expansion. Using this formulation the RGB values returned by a camera may be seen as a linear combination of integral basis functions so that $X = \sum_{n=0}^{\infty} k_n \int_{\lambda_0}^{\lambda_f} P(\lambda)b_n(\lambda)$.

The values of the expansion coefficients can then be determined by performing linear regression given a sufficient number of photographs with known spectra. For each of the three color channels the problem of obtaining the coefficients can be formulated as $\mathrm{argmin}_{\mathbf{k}} \|\boldsymbol{A}\mathbf{k} - \mathbf{X}\|_2$, where each row of the matrix $\boldsymbol{A}$ contains the result of the integrals of the spectrum of a single sample with the basis functions, $\mathbf{k}$ is the vector containing the expansion coefficients and $\mathbf{X}$ is a vector containing the true value of the color channel for that sample.

The manner in which a digital color camera converts a SPD into a triplet of RGB values depends on a variety of factors that are manufacturer, model, and user dependent. The transmissivity of the color filters of the camera Bayer array, the sensor sensitivity, the camera electronics, exposure time, and white balance all combine to form the set of CMFs that describe the camera's response to color. In order to ensure an accurate characterization of the CMF's, these factors must be kept static to the greatest possible extent. In this work we fix the camera parameters such that any automatic adjustments are disabled, and the exposure, gain and white balance are fixed to values that maintain the RGB values within the camera dynamic range without saturation or under-exposure for all samples.

Likewise, the point-to-point colors of a thin film depend on the local optical path length of the film, the illumination angle, the intensity of the light, and the color perception response of the observer. To simplify the description of the system, we observe the all samples under a Köhler illumination reflection microscope equipped with a camera (suppl. fig. 2a) and a constant power output light source. This fixes the angle of incidence of the input light as normal to the sample, the light intensity and the observer's color response.

In this work we characterize the camera's CMFs through the use of 65 thin film samples of known thickness and refractive index. Suppl. fig. 2b shows a photograph of a representative set of these samples, produced by spin coating polymethyl methacrylate (PMMA) photoresist to different sub-micron thickness on silicon wafer pieces. Suppl. fig. 2c shows the measured thickness of these samples. The color of each bar in the panel corresponds to the average RGB values the camera sees for the sample. Under the microscope the samples appear as uniform color objects, suppl. fig. 2d shows photographs of three examples seen at 100× magnification, alongside their measured and theoretical reflection spectra. We capture these image and spectra pairs for all calibration samples and then employ a linear regression process described earlier to recover the camera's CMFs.

Knowing the camera's CMFs we can compute the RGB triplet the camera will output for any SPD. The reflection spectra of a thin film can be analytically computed knowing its thickness and dispersion curve. While PMMA is dispersive from 400 nm to 700 nm, the refractive in-

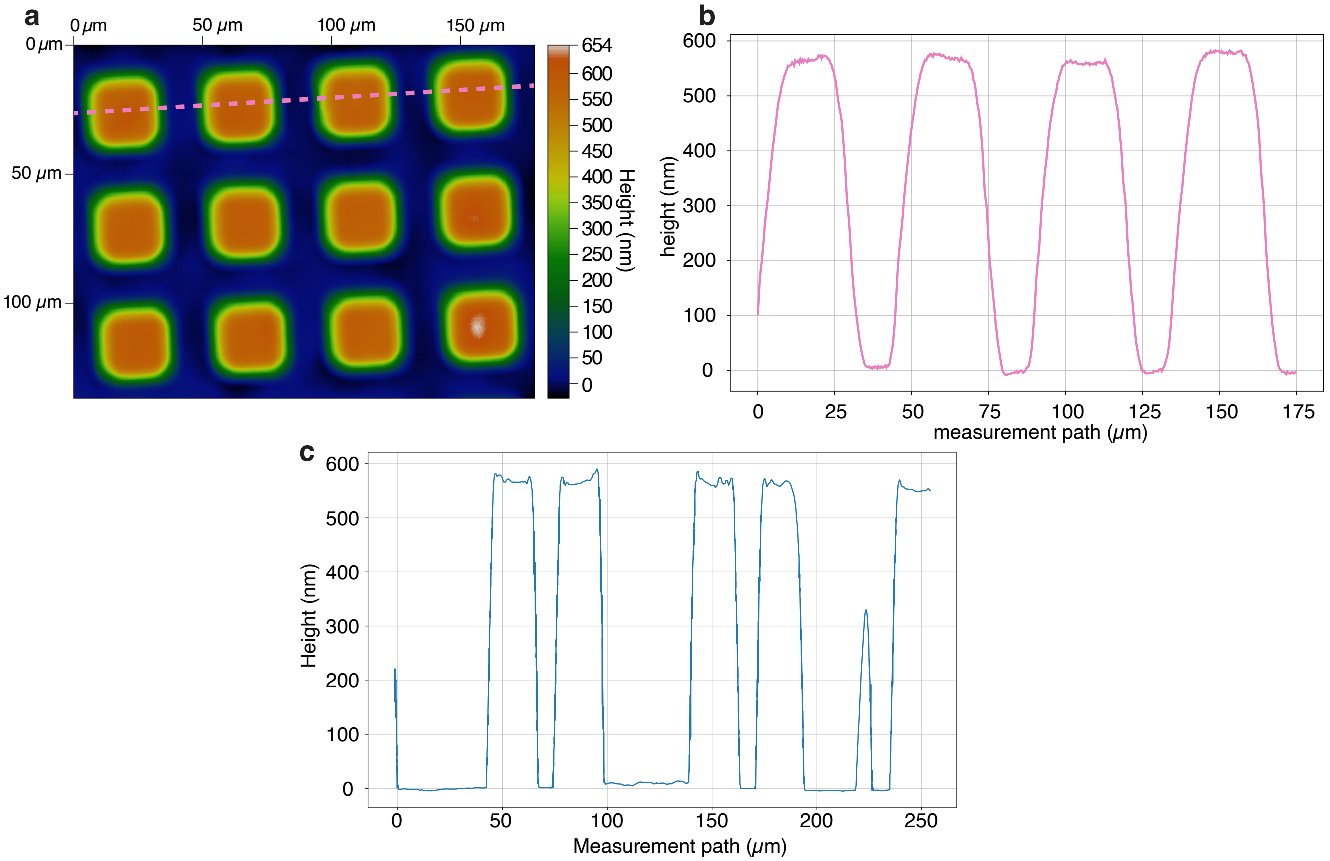

Supplementary Figure 1: Profilometer measurements of calibration cell's thickness. **a** Two dimensional optical profilometer thickness measurement of a group of synthetic cells prior to the removal of the AZ1505 suporting resist layer. **b** 1D height profile corresponding to the dashed pink line in panel **a**. **c** 1D contact profilometer measurement of the cells shown in panel **a**. The x axis of the plot corresponds to the distance travelled by the profilometer cantilever over the wafer surface, while the y axis shows the recorded height.

dex variation is smaller than the 10^{-2} sensitivity we can achieve with the 12 bit camera used in this work (See Fig. 3 in the main text) [1]. We therefore consider the average value of the PMMA refractive index in this range for our computations. We numerically compute a set of reflection spectra for different refractive index and thickness thin films on silicon, and compute the color the camera will output upon seeing them. This allows us to generate a lookup table (suppl. fig. 2e) that maps refractive index and thickness pairs for the calibration thin films to RGB triplets. Suppl. fig. 2f presents the lookup table for biological material thin films, which we obtain through an analogous procedure to the one used for suppl. fig. 2e.

This technique requires the appearance of thin film interference colors from the sample. The visibility of the colors decreases when the sample's thickness is significantly larger than the wavelength. Suppl. fig. 2e-f show this trend on the far right of the plots. Increasing the measurement range requires increasing the source's coherence length while ensuring a sufficiently broad spectral coverage.

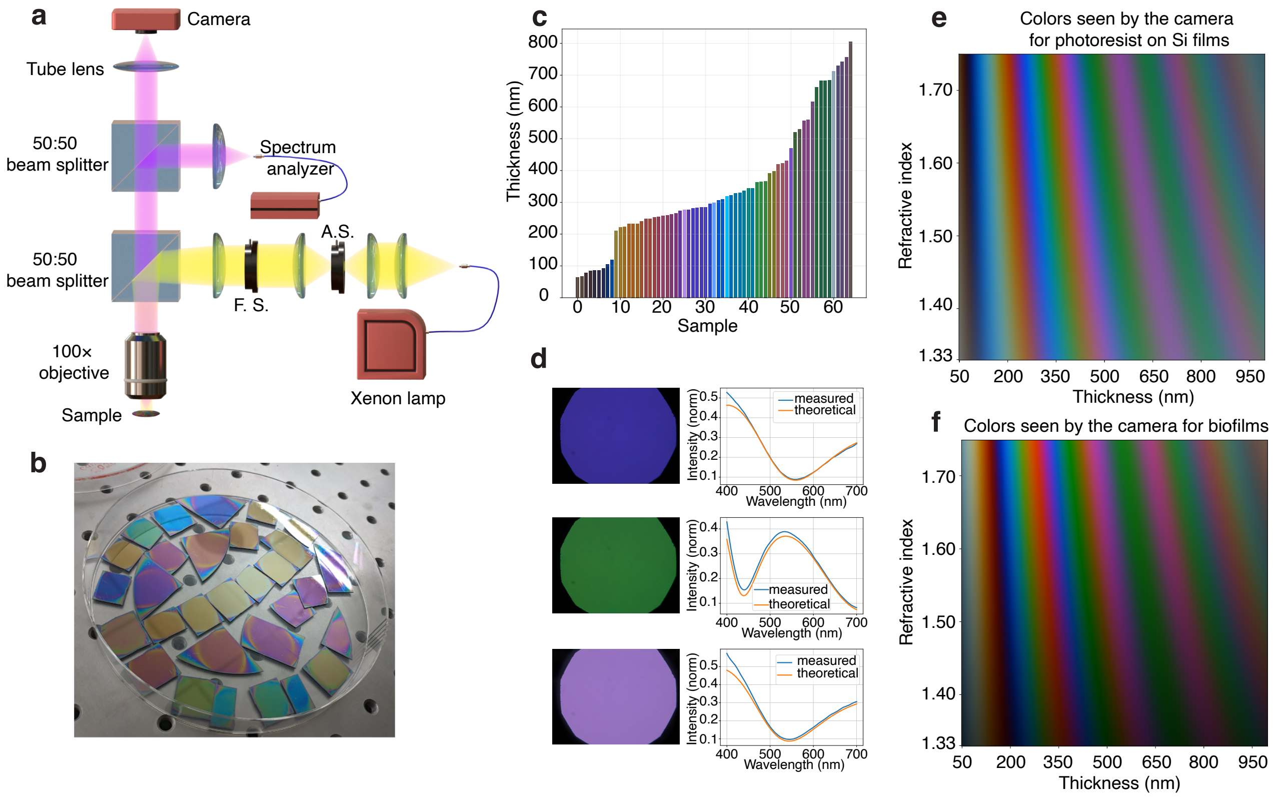

Supplementary Figure 2: Optical setup and calibration a. The measuring setup consists of a reflection microscope setup with Köhler illumination. An additional beam splitter is used in conjunction with a spectrum analyzer to capture the reflection spectra of the calibration samples but is not required for the cell analysis. Panel **b** shows a photograph of the calibration samples, these consist of PMMA photoresist spin coated to different thicknesses on silicon wafer pieces. Panel **c** shows the measured thickness for all calibration samples, the color of each bar corresponds to the average color of the sample. Panel **d** shows examples of the images seen through the setup for three calibration samples, alongside their measured and theoretical reflection spectra. Panel **e** shows the lookup table of thickness and refractive index to RGB for PMMA thin films seen by the camera. Panel **f** shows the lookup table of thickness and refractive index to RGB for biological thin films seen by the camera.

Supplementary References

1. Zhang, X., Qiu, J., Li, X., Zhao, J. & Liu, L. Complex Refractive Indices Measurements of Polymers in Visible and Near-Infrared Bands. *Applied Optics* **59,** 2337 (Mar. 10, 2020).
