# Supplementary material for: Real-time simultaneous refractive index and thickness mapping of sub-cellular biology at the diffraction limit
Source: Commun Biol. 2024 Feb 6;7:154. doi: 10.1038/s42003-024-05839-w (PMC10847501; doi:10.1038/s42003-024-05839-w)
Supplement: Supplementary file 3 — Reporting Summary [file 42003_2024_5839_MOESM3_ESM.pdf]

nature portfolio

Corresponding author(s): Andrea Fratalocchi

Last updated by author(s): Jan 2, 2024

Reporting Summary

Nature Portfolio wishes to improve the reproducibility of the work that we publish. This form provides structure for consistency and transparency in reporting. For further information on Nature Portfolio policies, see our Editorial Policies and the Editorial Policy Checklist.

Statistics

For all statistical analyses, confirm that the following items are present in the figure legend, table legend, main text, or Methods section.

n/a	Confirmed	
☒	☐	The exact sample size (*n*) for each experimental group/condition, given as a discrete number and unit of measurement
☒	☐	A statement on whether measurements were taken from distinct samples or whether the same sample was measured repeatedly
☒	☐	The statistical test(s) used AND whether they are one- or two-sided<br>*Only common tests should be described solely by name; describe more complex techniques in the Methods section.*
☒	☐	A description of all covariates tested
☒	☐	A description of any assumptions or corrections, such as tests of normality and adjustment for multiple comparisons
☒	☐	A full description of the statistical parameters including central tendency (e.g. means) or other basic estimates (e.g. regression coefficient) AND variation (e.g. standard deviation) or associated estimates of uncertainty (e.g. confidence intervals)
☒	☐	For null hypothesis testing, the test statistic (e.g. *F*, *t*, *r*) with confidence intervals, effect sizes, degrees of freedom and *P* value noted<br>*Give P values as exact values whenever suitable.*
☒	☐	For Bayesian analysis, information on the choice of priors and Markov chain Monte Carlo settings
☒	☐	For hierarchical and complex designs, identification of the appropriate level for tests and full reporting of outcomes
☒	☐	Estimates of effect sizes (e.g. Cohen's *d*, Pearson's *r*), indicating how they were calculated

Our web collection on statistics for biologists contains articles on many of the points above.

Software and code

Policy information about availability of computer code

Data collection	OceanView- 1.6.7 - Commercial spectrum analyzer operation software from Ocean Insight used for recording the reflection spectra of the calibration samples.<br>Thorcam - 3.7.0 - Commercial image acquisition software for Thorlabs cameras. Used for taking all sample pictures under the microscope.
Data analysis	All analysis were carried out using Python 3.9.13 with the following freely available libraries:<br>numpy 1.21.5, imageio 2.19.3, matplotlib 3.5.2, joblib 1.1.0, scikit-learn 1.0.2, scipy<br>1.9.1, tmm 0.1.8, colour-science 0.4.1, tqdm 4.64.1, regex 2022.7.9, jupyterlab 3.4.4<br>All custom algorithms used are available at the following GitHub repository: https://github.com/burguea/biocolors

For manuscripts utilizing custom algorithms or software that are central to the research but not yet described in published literature, software must be made available to editors and reviewers. We strongly encourage code deposition in a community repository (e.g. GitHub). See the Nature Portfolio guidelines for submitting code & software for further information.

Data

Policy information about availability of data

All manuscripts must include a data availability statement. This statement should provide the following information, where applicable:

- Accession codes, unique identifiers, or web links for publicly available datasets
- A description of any restrictions on data availability
- For clinical datasets or third party data, please ensure that the statement adheres to our policy

All data supporting the findings of this study is available via the repository: https://github.com/burguea/biocolors

Human research participants

Policy information about studies involving human research participants and Sex and Gender in Research.

Reporting on sex and gender	The findings of this study do not depend on sex or gender. While the study made use of the HCT-116 cell line, which was originally isolated from a human male, the results hold for cells isolated from humans of any sex or gender as the method depends only on the intrinsic physical properties of microorganisms.
Population characteristics	The manuscript does not use any socially constructed or socially relevant variables.
Recruitment	This study does not make use of human research participants.
Ethics oversight	This study did not require approval by an ethics committee.

Note that full information on the approval of the study protocol must also be provided in the manuscript.

Field-specific reporting

Please select the one below that is the best fit for your research. If you are not sure, read the appropriate sections before making your selection.

☒ Life sciences ☐ Behavioural & social sciences ☐ Ecological, evolutionary & environmental sciences

For a reference copy of the document with all sections, see nature.com/documents/nr-reporting-summary-flat.pdf

Life sciences study design

All studies must disclose on these points even when the disclosure is negative.

Sample size	No sample size calculation was performed. The aim of this study is to present an imaging technique, not to investigate a population.
Data exclusions	No data was excluded from the analysis.
Replication	Over 50 samples of nanostructured Pd were produced during this study across several months with the intent of optimizing the electrodeposition parameters for broadband light absorption. HCT-116 cells originating from over 10 independent cultures were deposited on each of these samples during this time.<br>The phenomenom of structural coloration was observed on all attempts in which the Pd samples were produced according to the parameters described in the manuscript (>10 samples).
Randomization	Randomization is not relevant to this study. The aim of this study is to present an imaging technique, not to investigate a population.
Blinding	Blinding is not relevant to this study. The aim of this study is to present an imaging technique, not to investigate a population.

Reporting for specific materials, systems and methods

We require information from authors about some types of materials, experimental systems and methods used in many studies. Here, indicate whether each material, system or method listed is relevant to your study. If you are not sure if a list item applies to your research, read the appropriate section before selecting a response.

Materials & experimental systems

n/a	Involved in the study
☒	☐ Antibodies
☐	☒ Eukaryotic cell lines
☒	☐ Palaeontology and archaeology
☒	☐ Animals and other organisms
☒	☐ Clinical data
☒	☐ Dual use research of concern

Methods

n/a	Involved in the study
☒	☐ ChIP-seq
☒	☐ Flow cytometry
☒	☐ MRI-based neuroimaging

Eukaryotic cell lines

Policy information about cell lines and Sex and Gender in Research

Cell line source(s)	HCT 116 cells belong to a cell line of colon cancer cells isolated from the colon an adult human male. The cells in this study were purchased from ATCC (product CCL-247TM)
Authentication	ATCC Cell Line Authentication Service<br>Sanger Sequencing
Mycoplasma contamination	Cells tested negative for mycoplasma contamination
Commonly misidentified lines (See ICLAC register)	No commonly misidentified lines were used.
